# Prospective decision making for randomly moving visual stimuli

**DOI:** 10.1038/s41598-019-40687-3

**Published:** 2019-03-07

**Authors:** Ryuto Yashiro, Hiromi Sato, Isamu Motoyoshi

**Affiliations:** 1 Department of Life Sciences, The University of Tokyo, 3-8-1 Komaba, Meguro-ku, Tokyo 153-8902 Japan; 2 Faculty of Informatics, Kogakuin University, 1-24-2 Nishi-shinjuku, Shinjuku-ku, Tokyo 163-8677 Japan; 3 JSPS Research Fellow, Tokyo, Japan

## Abstract

Humans persist in their attempts to predict the future in spite of the fact that natural events often involve a fundamental element of uncertainty. The present study explored computational mechanisms underlying biases in prospective decision making by using a simple psychophysical task. Observers viewed a randomly moving Gabor target for T sec and anticipated its future position ΔT sec following stimulus offset. Applying reverse correlation analysis, we found that observer decisions focused heavily on the last part of target velocity and especially on velocity information following the last several direction reversals. If target random motion explicitly contained an additional linear trend, observers tended to utilize information of the linear trend as well. These behavioral data are well explained by a leaky-integrator model of perceptual decision making based on evidence accumulation with adaptive gain control. The results raise the possibility that prospective decision making toward future events follows principles similar to those involved in retrospective decision making toward past events.

## Introduction

Predicting the future state of events is crucial for adaptive behaviors in nature and society. We continually attempt to predict future events of various temporal scales such as the trajectory of a tennis ball, the stock market, the weather, and catastrophic disasters. Future events are full of uncertainty, and mankind has correspondingly developed skills and technologies to predict future events as accurately as possible. However, we have little knowledge about how the individual brain predicts the future at large temporal scales.

The sensory system is capable of predicting the short-range future state of dynamic inputs if these inputs contain regularities. For instance, the visual system implicitly predicts incoming inputs by utilizing the spatiotemporal regularities in natural images and movies^1,2^. Such predictive coding is so robust that it can sometimes produce systematic errors in perception under specific circumstances; i.e., illusions^3–6^. Yet, this predictive system works successfully only over a range of several hundred milliseconds^7^.

Humans and animals are able to predict the state of a stimulus several seconds into the future if the stimulus is governed by simple and explicit rules. For instance, we can perceive the rhythm in a sequence of periodical sounds^8–10^ and accurately respond to the next stimulus in the sequence^11–13^ by consciously or unconsciously learning rules governing the stimulus^14–16^. In the real world, however, most events are inherently uncertain (chaotic or truly random) particularly at large temporal scales. It would be impossible for us to predict such uncertain futures accurately, and we are in fact very poor at predicting the evolution of a state – the location of flying birds, the price of a stock, and so on – a few seconds beyond the present time. Nevertheless, we often take predictive decisions and actions toward such unpredictable future events regardless of whether our decisions succeed or not.

Little is known about the information processing that underlies erroneous prospective behaviors toward unpredictable events. Studies in the disciplines of behavioral economics and social psychology have revealed that human prospective decision-making applied to uncertain events (which are not necessarily about the future) is dominated by systematic biases^17–19^. However, the findings from these fields are primarily based on responses to stimuli given as sentences and scenarios and hence involve complex problems associated with neural processing of language and logic.

By comparison, several psychophysical studies analyze human predictive responses by using a simple and physically defined visual stimulus. For example, humans are able to estimate visual and motion uncertainty to extrapolate the future location of a moving dot with stochastic variability, whether or not a reward is given^20,21^. Although these studies clearly indicate how sensitive humans are to endogenous and exogenous uncertainty when making predictions, the temporal dynamics underlying such predictive actions has not yet been fully characterized. The present study quantitatively investigates temporal dynamics of prospective behavior within the framework of perceptual decision making^22^. To this end, we applied reverse correlation analysis which enables the characterization of the system’s response to arbitrary inputs in a neutral fashion^23^. Reverse correlation is a tool that has long been used in neuroscience and psychophysics of perception^24–26^ and in recent studies of decision making toward stochastic visual stimuli^27–29^. By applying reverse correlation to responses made to randomly moving visual stimuli, we explored computational principle underlying human prospective decision making about the future of dynamic events.

## Results

**Figure 1:**
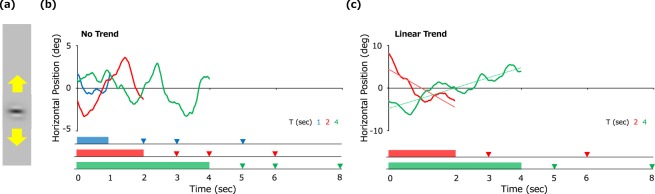
(**a**) Visual stimulus. (**b**) Curves show motion trajectories of the stimulus in the first experiment with duration (T) of 1 sec (blue), 2 sec (red) and 4 sec (green), respectively. Triangles at the bottom represent times (ΔT) at which observers were asked to predict the stimulus position. (**c**) Curves show motion trajectories of the stimulus in the second experiment with duration (T) of 2 sec (red) and 4 sec (green), respectively. The dashed lines are linear trends of the trajectories.

Twelve human observers viewed a Gabor pattern moving horizontally according to a velocity noise profile that followed a 1/f frequency spectrum with a low-frequency cut-off (Fig. 1a). The stimulus was presented for a duration (T) of 1, 2, or 4 sec (Fig. 1b). Following stimulus offset, observers indicated whether the stimulus will be located to the left or to the right of the offset location after a particular time lag (ΔT) of 1, 2, or 4 sec (denoted by triangles in Fig. 1b). In other words, observers anticipated toward which direction the stimulus would move over the ΔT interval from stimulus offset. Observers were asked to assume that the same pattern of stimulus movement would continue following stimulus offset and to respond within 0.5 sec. T and ΔT were held constant in each experimental block.

As the observers constantly attempt to predict the future location of the stimulus, responses should clearly reflect a particular strategy and bias in decision making about future stimulus location. To elucidate how observers utilize stimulus information during presentation, we calculated the logistic regression coefficient of stimulus velocity for each temporal frame of the observer’s response. This coefficient can be thought of as the “impact” of the velocity at each temporal frame upon the observer’s decision making^30^. A higher impact for a particular frame signifies that an observer is giving more weight to velocity information at that frame in making decision.

**Figure 2:**
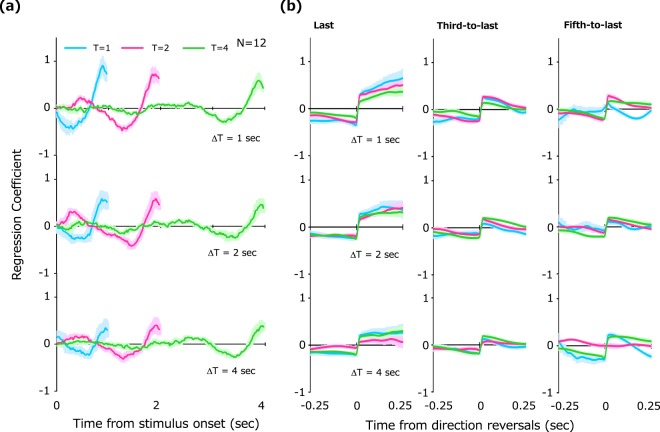
Reverse correlation analysis of prospective responses to the randomly moving stimulus. (**a**) Regression coefficients (impacts) of stimulus velocity plotted as a function of time from stimulus onset. Different curve colors represent results for different durations (T). Each panel shows results for different lags (ΔT). (**b**) Impacts of stimulus velocity on and around direction reversals. The results for last- (left), third-to-last (middle) and fifth-to-last (right) reversals are shown. Shaded regions around the curves represent + −1SE across observers.

Figure 2a shows the impact of the velocity plotted as a function of temporal frame. Each color represents the duration T, and each panel shows the results for each lag ΔT. All three panels show that impact is especially high just before the stimulus offset (a recency bias) and that this bias becomes less marked as ΔT is made longer. Note that the negative impact before the positive peak is an artifact of the velocity waveform’s autocorrelation (see Methods). A three-way repeated measures ANOVA on average impact was performed with factors of T, ΔT, and temporal epoch (10 frames just before the stimulus offset vs. from stimulus onset to the 10th frame to last). There was a main effect of ΔT (*F*(2, 22) = 7.23, *p* = 0.004), a main effect of epoch (*F*(1, 11) = 12.4, *p* = 0.005), and an interaction between ΔT and epoch (*F*(2, 22) = 7.93, *p* = 0.003). No significant main effect of T was observed (*F*(2, 22) = 0.92, *p* = 0.41). A multiple-comparison Tukey test revealed that average impacts over 10 frames just before stimulus offset was significantly higher for ΔT = 1 sec than for ΔT = 4 sec (*t*(11) = 1.25, *p* = 0.03). There was no significant difference between impact averages for ΔT = 1 sec and ΔT = 2 sec (*t*(11) = 0.95, *p* = 0.09), and between averages for ΔT = 2 sec and ΔT = 4 sec (*t*(11) = 0.97, *p* = 0.09). These results clearly suggest that observers judge the future location of the randomly moving stimulus by relying on the last part of the available information regardless of how long they received stimulus information and that the effect of recent-information emphasis becomes weaker as observers are asked to predict further into the future.

Our results raise the question of what aspects of the last information segment observers emphasized in particular. Given that the visual system is highly sensitive to changes, one possible aspect that greatly contributes to decision making is the moment of the last direction reversal. Here, ‘direction reversal’ is taken to refer to the physically defined and perceptible moment at which stimulus motion switches to the opposite direction. This notion of direction reversal leads us to a further possibility that information following the last direction reversal plays an especially important role in the final judgment on future stimulus movement. To examine this possibility, we calculated impacts during 0.25 sec before and after the last three direction reversals. These analyses were applied on velocity waveforms that were slightly blurred by a Gaussian filter with a time constant of 33 ms in order to eliminate extremely small reversals. Note that the motion of the stimulus with the shorter duration contained fewer direction reversals. In fact, the number of direction reversals was less than 5 in half of the trials if stimulus duration was as short as 1 sec, but the number of the data collected for three reversals was nonetheless still sufficient for our analysis.

Figure 2b shows the impact of stimulus velocity during 0.25 sec before and after the three last reversals – impact rises sharply immediately after direction reversals. This rapid increase is most remarkable after the last reversal. Statistical significance was assessed with a two-way repeated measure ANOVA followed by a multiple comparison test (Tukey test). There was a significant main effect of the period (before vs. after) relative to the reversal (*F*(1, 11) = 16.1, *p* = 0.002), temporal order of the reversal (*F*(2, 22) = 8.70, *p* = 0.002), and a significant interaction between these two factors (*F*(2, 22) = 18.3, *p* < 0.001). The following analysis revealed significant differences of average impacts after the reversal between the last vs. the third-to-last (*t*(11) = 1.64, *p* = 0.005), and the last vs. the fifth-to- last (*t*(11) = 1.64, *p* = 0.005), but not the third to last vs. the fifth to last direction reversal (*t*(11) = 0.60, *p* = 0.31). These analyses indicate that decisions were made with emphasis on information just after the last several direction changes, especially on information immediately following the last reversal.

The results above demonstrate that observers utilize only recent inputs to make prospective decisions for randomly moving stimuli. However, observers may make use of explicit regularities and alter prediction strategy if such regularities are available in the stimulus. For example, observers may predict the future state of a stimulus simply by extrapolating a linear trend if such a trend was contained in the stimulus. To explore this possibility, we employed 1/f velocity-noise stimuli and added a linear trend (i.e., constant additional velocity) (Fig. 1c). We used the linear trend because it is the simplest one. The amount of additional velocity was randomly determined on each trial according to a Gaussian distribution with a particular SD (4.2 deg/sec for T of 2 sec and 2.1 deg/sec for T of 4 sec). Using the same procedure as above, we asked observers to anticipate stimulus location ΔT sec (1 or 4 sec) after stimulus offset and to assume that the same pattern of stimulus movement would continue.

**Figure 3:**
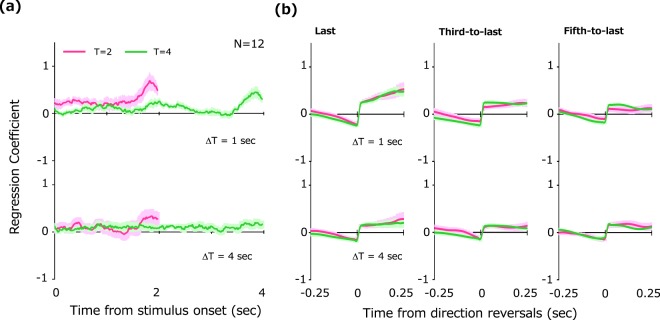
Reverse correlation analysis of prospective responses for stimuli containing linear velocity trends. (**a**) Regression coefficients (impacts) of stimulus velocity plotted as a function of time from stimulus onset. Different curve colors represent results for different durations (T). Each panel shows results for different lags (ΔT). (**b**) Impacts of stimulus velocity on and around direction reversals. The results for last- (left), third-to-last (middle) and fifth-to-last (right) reversals are shown. Shaded regions around the curves represent + −1SE across observers.

As in the previous experiment, we calculated the impact of the stimulus velocity for each temporal frame. The results in Fig. 3a show a tendency similar to that of previous data for stimuli without linear trends. A three-way repeated measure ANOVA on average impact revealed a significant main effect of ΔT (*F*(1, 11) = 8.07, *p* = 0.02), temporal epoch (*F*(1, 11) = 7.99, *p* = 0.02), and interaction between ΔT and epoch (*F*(1, 11) = 6.36, *p* = 0.03). No significant main effect of T was observed (*F*(1, 11) = 3.86, *p* = 0.08). There was a significant difference of average impacts over 10 frames just before stimulus offset (*t*(11) = 1.38, *p* = 0.006, Tukey test). Even with stimuli containing a linear trend, observers still emphasized recent change in information, but this recency bias became less profound as observers were asked to predict further into the future.

Results in Fig. 3a show the unique property that impacts always maintain a positive value (ie, larger than zero) in the early part of stimulus presentation. This is not observed in the previous experiment where linear trends were absent. To test the effect of linear trend on these constant impacts, we compared average impact between experiments with and without linear trends by computing impact over all frames but the last 10. A three-way repeated measure ANOVA with T, ΔT, and the absence/presence of linear trends revealed a significant main effect of linear trend (*F*(1, 11) = 4.54, *p* = 0.05). No significant main effect of T (*F*(1, 11) = 0.98, *p* = 0.34) and ΔT (*F*(1, 11) = 1.23, *p* = 0.29) were observed. These results show that observers exploited linear stimulus trends in making decisions about the future.

As in the previous experiment, impacts surge immediately after direction reversals (Fig. 3b). We again conducted a two-way repeated measure ANOVA on average impact and observed a significant main effect of period relative to reversal (*F*(1, 11) = 19.4, *p* = 0.001), temporal order of reversal (*F*(2, 22) = 3.57, *p* = 0.04), and a significant interaction between these two factors (*F*(2, 22) = 10.2, *p* < 0.001). There was a significant difference revealed by Tukey test between the last- vs. third-to-last (*t*(11) = 1.24, *p* = 0.02) and the last vs. the fifth-to-last (*t*(11) = 1.43, *p* = 0.01), but not the third-to-last vs. the fifth-to-last direction reversals (*t*(11) = 0.66, *p* = 0.28), thereby indicating that decisions were made with emphasis especially on information immediately following the last several reversals.

**Figure 4:**
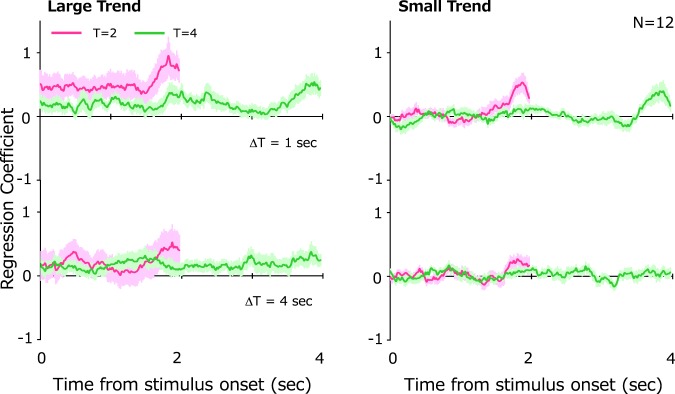
Impact curves for stimuli with large trends (left) and small trends (right). Conventions are all the same as in Fig. 3.

In this experiment, the magnitude of the linear trend was randomly varied across trials. The trend was clearly visible to observers in some trials but not in others. The constant additional impact obtained above might result from a different strategy based on the observers’ knowledge that stimulus motion sometimes contained linear trends. If such is the case, then the additional impact should be evident regardless of linear trend magnitude. To examine this possibility, we calculated impact separately for trials categorized into large and small linear trends on the basis of a median split. The results in Fig. 4 show that the constant additional impact is found only for trials with larger trend. The average impact throughout the presentation was submitted to a three-way repeated measure ANOVA with factors of ΔT, T, and trend magnitude. The analysis revealed that a main effect of trend magnitude approached significance (*F*(1, 11) = 3.91, *p* = 0.07). There was no significant main effect of ΔT (*F*(1, 11) = 2.20, *p* = 0.16) and T (*F*(1, 11) = 2.71, *p* = 0.12). These results indicate that observers utilized trend information simply because it was available.

## Discussion

To understand mechanisms of human prospective decision making toward uncertain future events, the present study carried out a psychophysical experiment in which human observers anticipated the future location of a visual stimulus undergoing a quasi-random walk. By means of reverse correlation analysis between stimulus motion and observer response, we found that, in many conditions, observers made decisions about the future by relying heavily on recent-change information and especially on information delivered after the last several direction reversals. The results also showed that humans can often make use of simple linear stimulus motion trends in making predictions provided that observers can perceive the trends.

The observed recency bias would seem to contradict a previous study showing that humans accumulate visual information over a long period of time^20^. However, unlike the previous study, it should be noted that stimulus motion in our first experiment did not contain any explicit trend (Fig. 1b). It is therefore reasonable for observers to depend solely on recent information in this particular case. In the second experiment, our stimuli included a linear-trend motion component (Fig. 1c), and observers utilized the overall trend, in line with the previous study cited above (Fig. 4).

What type of computations gave rise to behaviors characteristic of our results? Had observers anticipated the future simply by integrating all available information equally over time, then the impact curve would have been completely flat. In actuality, however, we found a remarkable peak around stimulus offset, which is a clear manifestation of the recency bias. Interestingly, such a recency bias has been widely reported in studies of perceptual (i.e., retrospective) decision making for dynamic noisy stimuli^28,31–33^. Indeed, prospective decision may build on similar general principles whereby the brain integrates noisy inputs over time to make decision.

A number of psychophysical primate experiments have shown that perceptual decision making can be typically described by a fundamental mechanism that linearly accumulates evidence over time and makes a decision when the amount of evidence reaches a decision boundary^22,34,35^. The Drift Diffusion Model (DDM) is one such theory of linear accumulation of decision-relevant information. DDM accounts for distributions of response time and error rate^36,37^, and the validity of DDM has been underpinned by time-dependent ramping of neural firing rates that are believed to reflect evidence accumulation^38–40^. The concept of linear evidence accumulation has been generally accepted as a computational framework of perceptual decision making. In line with these findings, Cheadle *et al*. (2014) recently developed a computational model in which decision-relevant information is linearly integrated over time, and the author’s model successfully replicated the recency bias in temporal-average estimation^41^. Importantly, in Cheadle’s model, decision-relevant information is generated by a transducer function that shifts constantly according to local information. That is, the model computationally exhibits the adaptive property of gain control found in primary visual cortex neurons whereby the contrast-response function shifts laterally so that a neuron’s limited dynamic range can nonetheless respond to a wide range of luminance or contrast^42^.

**Figure 5:**
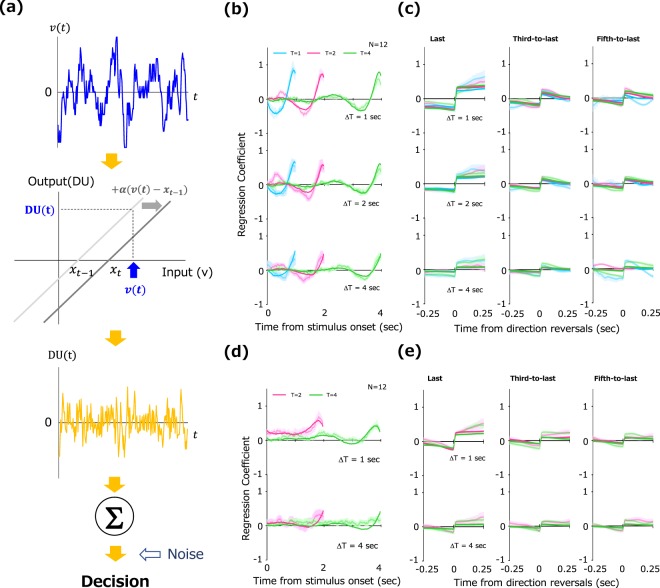
(**a**) An overview of our gain control model. An input (velocity) is converted into a decision update (DU) by a linear transducer function. The input gain of the function adaptively shifts according to preceding inputs. After repeating this operation throughout the presentation, the final decision is made based on the sum of DU over all temporal frames. (**b–e**) Impact curves predicted by the gain control model (solid lines) and human data (faint lines, replot of Figs 2 and 3) for stimuli without trends (**b,c**) and with linear trends (**d,e**).

Here, we simplified the adaptive gain control model by Cheadle *et al*. (2014) and tested whether a gain control model can replicate our data on prospective decision making. In our model, we define decision-relevant information as the decision update (DU). DU is assumed to be a linear product of stimulus velocity at each temporal frame t.

1 $${\rm{DU}}({\rm{t}})=v(t)-{x}_{t-1}$$

where *x*_*t−1*_ is the centroid of the function. The location of the centroid is shifted according to equation (2) described below. As shown in the middle panel of Fig. 5a, this corresponds to a parallel shift of the function along the horizontal axis. Such a shift is analogous to the gain control in the response function of retinal and cortical neurons^43,44^.

2 $${x}_{t}={x}_{t-1}+\alpha (v(t)-{x}_{t-1})$$

The amount of shift depends on the learning rate *α*. This effective update enables an adaptive behavior of the model since the output (DU) is continually adjusted by preceding inputs. The logit of final decision is determined by the sum of DU over all temporal frames and the scaling factor *β*.

3 $${\rm{logit}}(p)=\beta \sum _{t=0}^{T}{\rm{DU}}({\rm{t}})+\varepsilon $$

Using stimulus inputs identical to those used in the experiment, we sought to simulate impact curves from the response of the gain control model with two free optimized parameters (see Method). We searched the best fitting parameters for each ΔT, combining data over different T. The solid lines in Fig. 5b–e show the best predicted impact curves by the model, and the faint lines show human data (i.e., replots of Figs 2 and 3). The model impact curves replicate characteristic features observed in human data: noticeable peaks around stimulus offset and a sharp increase at the moment of reversals. We can also see the constant positive impacts in the linear-trend condition. The model data showed a good fit to human data (No trend: *χ*^2^ < 48.5; Linear trend: *χ*^2^ < 46.0) with estimated parameters of [*α*, *β*] = [0.14, 0.04] where ΔT was 1 sec, [*α*, *β*] = [0.25, 0.05] where ΔT was 2 sec, and [*α*, *β*] = [1.61, 0.18] where ΔT was 4 sec under the condition without trend, and [*α*, *β*] = [0.03, 0.01] where ΔT was 1 sec, [*α*, *β*] = [1.81, 0.17] where ΔT was 4 sec under the condition with the linear trend.

There is a mathematical reason why the gain control model produces the recency bias. Repetitive use of equations (1) and (2) results in the following equation:

4 $${\rm{logit}}(p)=\beta \sum _{t=0}^{T}{(1-\alpha )}^{T-t}v(t)+\varepsilon $$

Equation (4) quantitatively indicates that bigger weights are given to the later inputs (velocity) and smaller weights to the earlier inputs. This characteristic is also found in a class of leaky-integration decision models that predict the recency bias^28,32^. This similarity is reasonable given that leaky temporal integration is one of natural consequences of rapid gain control^44,45^ although leaky temporal integration could be produced by other mechanisms as well^7^. Models that do not include an adaptation component, including those that integrate decision-relevant information monotonously or use a Markov process, do not fully replicate the recency bias^30,46^. All evidence considered, the recency bias is therefore a likely product of leaky temporal integration of visual information as mediated by gain control.

It is noteworthy that the gain control model also captures almost the same period of evidence accumulation, for which impacts are above zero, across different lag times (see Fig. 5b and d). This can be also attributed to Equation (4), which illustrates that, regardless of ΔT, input weights decrease exponentially and approach zero as a function of *t*. This mathematical characteristic results in the similar, albeit different in amplitude, because of *α* in each condition, psychophysical kernels across the lag time.

The qualitative similarities between observers and the model highlight the validity of evidence accumulation with gain control as an underlying mechanism of human predictive behaviors. From a functional viewpoint, gain control enables the visual system to continually track changes in incoming sensory signals, and this operation has been argued to reflect the adaptive nature of predictive coding of information in the environment^47^. Thus, both prospective and retrospective (i.e., perceptual) decision makings seem to follow similar adaptive principles with respect to evidence accumulation.

Given the similarity between prospective and retrospective decision making at the behavioral level, there is a possibility that the two types of decision making share a common neural substrate. In studies on retrospective decision making, neurons in LIP and MT have been shown to integrate time-variant information to mediate decisions between saccadic responses^48,49^. Recently, psychophysical experiments in conjunction with EEG measurement show that decision-relevant information is continuously encoded in parietal cortex^41,50^. According to these findings, it might be the case that evidence accumulation preceding prediction about the future is also represented as neural activity over parietal cortex. Although the causal role of these regions in perceptual decision making has been recently challenged^51–53^, commonality in neural substrates between prospective and retrospective decision making is an intriguing problem that should be further explored in future studies.

The leaky-integrator property of the gain control model naturally lends itself to the successful reproduction of the sharp increase at the moment of the reversals. Strictly speaking, however, a small but significant gap is found between observed and predicted impacts after reversals (Fig. 5c and e). This gap not only suggests that the model underestimates the impacts after reversals but also emphasizes that humans have unique strategies that specifically focus on information after direction reversals when making prospective decisions. This strategy could be adaptive especially under a changeable environment where the later information might be more correlated with future events. While gain control is also adaptive for tracking changes in sensory signals, it is not necessarily optimal in our task where observers indicated the invisible future location of the stimulus instead of its sensory feature (e.g. the average velocity). Further, as stated above, recency bias can be obtained by leaky-integration models without gain control. These facts converge on the conclusion that evidence accumulation mediated by gain control is just one of the possible computational mechanisms for prospective decision making toward future events. Another model based on different principles could also account for the observed characteristics.

As is often the case with psychophysical findings obtained with simplified stimuli, it is unknown whether the present results are universally relevant to predictive behaviors (e.g., stock price prediction, ball trajectory anticipation, etc). It is possible that the recency bias is observable only for randomly moving visual stimuli. Consistent with this possibility are findings that the type of bias observed in retrospective decision making depends on the type of stimulus feature (e.g., drifting motion, discrete position, orientation, and faces, etc)^54^. It is therefore possible that we would have observed different patterns of results had we used different stimulus types (e.g., a discretely moving stimulus with sinusoidal trend, or digits presented sequentially). Nevertheless, we believe that the present study offers a basic framework for future investigations of prospective decision making that extends beyond the one-second duration regime.

Given that we presented stimuli with virtually no predictable trends (see Method), we could not define correct answers even probabilistically about future stimulus location. One possible concern about this stimulus design is that observers might actually respond randomly, and measured tendencies in such random responses would be totally irrelevant to observer decision. However, at least in the present study and probably in the previous studies with noisy stimuli, observers subjectively tried to predict the future as correctly as possible without being aware that a correct answer did not exist. Also note that the present psychophysical paradigm is largely consistent with numerous studies employing noisy motion displays (sometimes with no signal) to elucidate biases in perceptual decision making^41,55,56^. Analyzing responses to noisy inputs would be one of many suitable methods for exploring the basic characteristic of both retrospective and prospective decision makings.

## Methods

### Apparatus

Visual stimuli were generated by a PC (DELL Precision T1700) and displayed on a LCD monitor (BenQ XL-2430T) with a 60 Hz refresh rate. Pixel resolution was 0.02 deg/pixel at a viewing distance of 100 cm and mean luminance was 62.5 cd/m^2^. All experiments were conducted in a dark room.

### Observers

Ten naïve paid volunteers and two of the authors (21–30 years old, median = 22) participated in the experiment. All observers had normal or corrected to normal vision. All the experiments followed the Declaration of Helsinki guidelines, and were conducted with a permission from the Ethics Committee of the University of Tokyo. All observers provided informed consent.

### Stimuli

**Figure 6:**
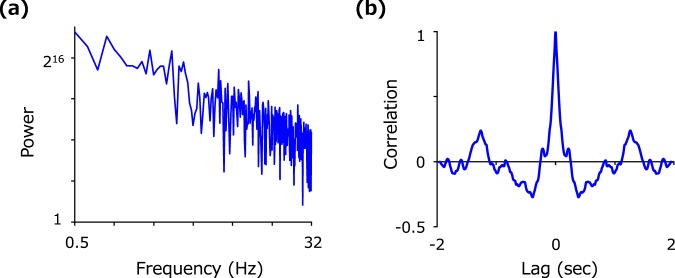
(**a**) Temporal frequency spectrum of the velocity noise used in the experiment. (**b**) The auto correlation profile of the velocity noise.

Visual stimuli consisted of a vertically oriented Gabor patch. The sinusoidal grating was presented as a circular patch with a diameter of 1.8 deg and a spatial frequency of 1.0 c/deg with a luminance contrast of 0.8. The stimulus changed its horizontal position along a virtual horizontal line 2.0 deg below the fixation point in accordance to random velocity noise. The velocity noise followed a 1/f frequency spectrum but temporal frequency components below 0.5 Hz were cut off to get rid of any explicit trends throughout the presentation. Figure 6 shows the temporal frequency spectrum of the velocity noise and the auto-correlation function. The auto-correlation function exhibits negative side lobes at a time lag of approximately 0.5 sec. It should be noted that this characteristic gives rise to negative lobes in the impact curve obtained from reverse correlation analysis; e.g., negative impacts immediately before the last positive impact in Fig. 2a. The stimulus was presented for a duration (T) of 1, 2, or 4 sec in each experimental block.

In the linear-trend condition, we added a linear velocity component to the velocity noise. This resulted in a globally gradual movement in one direction over the course of the stimulus presentation. Stimuli were presented for 2 or 4 sec. The magnitude of the additional velocity was determined on each trial following a Gaussian distribution with a particular SD (4.2 deg/sec for T of 2 sec and 2.1 deg/sec for T of 4 sec). It is important to note that observers could merely compare the onset and offset location to make decisions instead of constantly tracking changes in the location of the stimulus. However, the difference in SD resulted in the same average moving distance in two conditions for T, and this ensured that observers could not depend simply on the traveling distance of the stimulus when making decisions. Also, no observers actually reported making decisions based on that kind of strategy. Together, these facts show that observers could notice the trend in the movement of the stimulus only in some trials and basically made decisions by tracking the location of the stimulus.

### Procedure

Observers viewed the horizontal movement of the stimulus binocularly while fixating their gaze at the black fixation point in the center of the display. After the disappearance of the stimulus, observers indicated whether the stimulus will be located in the left or in the right of the offset location after a certain time lag (ΔT = 1, 2, or 4 sec for the stimulus without linear trend; ΔT = 1, 4 sec for the stimulus with linear trend) by pressing the appropriate key on a keyboard. That is, they attempted to predict the direction in which the stimulus will move over ΔT while holding on to the assumption that the same pattern of movement would continue into the future. To ensure that observers predicted future stimulus location - as opposed to postdicted - we explicitly asked observers to respond within 0.5 sec of the stimulus’ disappearance (i.e., always earlier than each ΔT). A tone was given just after the response if the response time exceeded 0.5 sec, and that trial was eliminated from further analysis. The next trial started 0.5 sec after the response. Both T and ΔT were held constant during each experimental session. Thus, there were 9 (3 × 3) conditions in total without linear trends, and 4 (2 × 2) conditions with linear trends. For each condition, observers completed a total of 240 trials on average.

### Analysis

We applied a logistic reverse correlation to examine how observers utilized velocity information on each temporal frame. In this method, we defined *p* as the probability of the observer responding “right”, which is formulated as a logistic function with two parameters: *a* and *b*. We then calculated the logistic regression coefficient (b) of the standardized velocity at each temporal frame t upon the observers’ response in each trial:

5 $${\rm{logit}}(p)=\,\mathrm{log}(\frac{p}{1-p})=a+b\cdot v(t)$$

The higher *b* in equation (5) at a particular temporal frame corresponds to higher observer sensitivity to stimulus velocity at that frame, that is, that particular velocity has a more significant influence on prospective decision making.

We also calculated impacts over the 0.25 sec before and after the last-, third-to-last, and fifth-to-last direction reversals. In order to eliminate extremely small reversals, the analysis was applied on velocity waveforms that were slightly blurred by a Gaussian filter with a time constant of 33 ms. The reason why we confined the analysis to every other reversal is that opposite direction of change between temporally adjacent reversals (e.g., the last and second-to-last reversal) results in opposite patterns of impacts - a natural property that is irrelevant to the discussion in the present study.

### Model simulation

We simulated impact curves predicted by the gain control model by fitting the model to average human data. The initial values of *α* and *β* were both set 1. The centroid *x*_*t*_ was initialized to 0 on each trial and was constantly updated according to equation (2) in the main text. The best-fitting values of the two-parameter model were determined without any constraint so that they minimized the mean squared error between the predicted and observed impacts at each temporal frame throughout the presentation. The model fitting was done for three (without linear trend) or two (with linear trend) different ΔT, and so we obtained three pairs of the best fitting parameters for the condition without any trend, and two pairs for the condition with the linear trend, as shown in Discussion. We also assessed the goodness of fit by calculating *χ*^2^ values formulated as follows:

6 $${\chi }^{2}=\sum _{t}\,\frac{{({b}_{t}-{\hat{b}}_{t})}^{2}}{{\sigma }_{t}^{2}}$$

*b*_*t*_ was the observed impact, ${\hat{b}}_{t}$ was the predicted impact by the model at each temporal frame and ${\sigma }_{t}^{2}$ was the variance of the observed impact.

## Data Availability

The datasets generated and analyzed during the current study are available from the corresponding author on reasonable request.
